# Supplementary material for: Cofactor Engineering for Efficient Production of α-Farnesene by Rational Modification of NADPH and ATP Regeneration Pathway in Pichia pastoris
Source: Int J Mol Sci. 2023 Jan 16;24(2):1767. doi: 10.3390/ijms24021767 (PMC9860691; doi:10.3390/ijms24021767)
Supplement: Supplementary file 1 [file ijms-24-01767-s001.zip › ijms-2020521-supplementary.pdf]

Supplementary materials

Cofactor engineering for efficient production of α-farnesene by rational modification of NADPH and ATP regeneration pathway in *Pichia pastoris*

Sheng-Ling Chen [1], Ting-Shan Liu [1], Han-Guang Li [2, *], Wei-Guo Zhang [1], Jian-Zhong Xu [1, *]

Supplementary_data_1:

Plasmid construction process

Ligation of fragments and vectors using the ClonExpress II One Step Cloning Kit (Vazyme Biotech, Nanjing, China). The fragments of homologous recombination include homologous fragments of 15-20 bp. The constructed plasmid was amplified using *E. coli* JM109. sgRNA design using http://chopchop.cbu.uib.no/ website.

Construction of strains

The gene integration and insertion of *Pichia pastoris* engineering strains were carried out by electroporation, and Cre/LoxP system was used for the recovery of screening resistance, in which the gene expressing Cre enzyme used an episomal plasmid, and antibiotic-free culture was used to eliminate the plasmid. The homologous fragment used for knockout and insertion in the CRISPR/CAS9 system is about 1000 bp.

Supplementary_data_2:

Table S1 Primers used for expression vectors in this study

Primers named	Primers（5'-3'）
PGAP-Z-F	TATGCTATACGAACGGTAATCATGTAATTAGTTATGTCACG
PGAP-Z-R	GGATCCGCACAAACGAAGGTCTCACTTAATCTTCTGTACTC
Lox71-F	TACCGTTCGTATAATGTATGCTATACGAAGTTATGGATCCCCCACACACCA TAG
Lox66-R	TACCGTTCGTATAGCATACATTATACGAAGTTATATTAAGGGTTCTCGAGA GCTC
HIS4-F	CTTCGTTTGTGCGGATCCTTAAATAAGTCCCAGTTTCTC
HIS4-R	CATACATTATACGAACGGTAATGACATTTCCCTTGCTACC
PIS1-F	GTCATGCATGAGATCAGATCGTAACGAGGCTAAAAGTTTTTGC
PIS1-R	GAATTCCTCGTTTCGAATGCAGGTGGACTATCTAGAGACAAG
PGPM1-F	TCATGCATGAGATCAGATC AAGGTTTATCTGACTGTTGCGCAC
PGPM1-R	GAATTCCTCGTTTCGATGTTTGTTTGTGTAATTGAAAGTT
PMET3-F	TCATGCATGAGATCAGATCAATTCAGGCAACAGGACC
PMET3-R	GAATTCCTCGTTTCGATTCTTTCTGAGTTGGTTTCC
PPGK1-F	TCATGCATGAGATCAGATCTACCCAGCCGATCACGCCTGC
PPGK1-R	GAATTCCTCGTTTCGAGGGCCCAAGTTGGTACCCAGCC
ZWF1-F	TCGAAACGAGGAATTCATGACCGATACGAAAGCCG
ZWF1-R	GGCCGCCGCGGCTCGAGTTACATCTTGTGCAGCACATC
sol3-F	TCGAAACGAGGAATTCATGGTACAAATCTATTCCT
sol3-R	CGGCCGCCGCGGCTCGAGTCAGTATTTCGAAGTAGAAAC
Gnd2-F	TCGAAACGAGGAATTCATGGTTGAAGCAACAGGAG

Gnd2-R	GGCCGCCGCGGCTCGAGTTAAGCATCGTAGGTACTGG
Rpel-F	TCGAAACGAGGAATTCATGGTTAAACCTATTATTGC
Rpel-R	GGCCGCCGCGGCTCGAGTCATAAGTTGGGTTTTGGTTTG
cPOS5-F	TCGAAACGAGGAATTCATGTTTGTCAGGGTTAAATTG
cPOS5-R	GGCCGCCGCGGCTCGAG TTAATCATTATCAGTCTGTCTC
APRT-F	TCGAAACGAGGAATTCATGTCAACCGCTTCTCTG
APRT-R	GGCCGCCGCGGCTCGAGTCATTTCTGCTGCCCCGA
PGI-sg	TGTTCACTTCGTATCCAACG
GPD1-sg	GTTGACGGATATTATCAACG
